# Simulational investigation of self-aligned bilayer linear grating enabling highly enhanced responsivity of MWIR InAs/GaSb type-II superlattice (T2SL) photodetector

**DOI:** 10.1038/s41598-024-52113-4

**Published:** 2024-01-24

**Authors:** Minseok Lee, Zahyun Ku, Seungjin Jeong, Jehwan Hwang, Junghyun Lee, Junoh Kim, Sang-Woo Kang, Augustine Urbas, Hagyoul Bae, Bongjoong Kim

**Affiliations:** 1 Department of Mechanical and System Design Engineering, Hongik University, Seoul, 04066 Republic of Korea; 2 Materials and Manufacturing Directorate, Air Force Research Laboratory, Wright-Patterson Air Force Base, Wright-Patterson AFB, 45433 USA; 3 Apex Microdevices, 45069 West Chester, USA; 4 Optical Lens Materials Research Center, Korea Photonics Technology Institute (KOPTI), Gwangju, 61007 Republic of Korea; 5 Advanced Instrumentation Institute, Korea Research Institute of Standards and Science, Daejeon, 34113 Republic of Korea; 6 Department of Electronic Engineering, Jeonbuk National University, Jeonju, 54896 Republic of Korea

**Keywords:** Engineering, Optics and photonics, Physics

## Abstract

Linear gratings polarizers provide remarkable potential to customize the polarization properties and tailor device functionality via dimensional tuning of configurations. Here, we extensively investigate the polarization properties of single- and double-layer linear grating, mainly focusing on self-aligned bilayer linear grating (SABLG), serving as a wire grid polarizer in the mid-wavelength infrared (MWIR) region. Computational analyses revealed the polarization properties of SABLG, highlighting enhancement in TM transmission and reduction in TE transmission compared to single-layer linear gratings (SLG) due to optical cavity effects. As a result, the extinction ratio is enhanced by approximately 2724-fold in wavelength 3–6 μm. Furthermore, integrating the specially designed SABLG with an MWIR InAs/GaSb Type-II Superlattice (T2SL) photodetector yields a significantly enhanced spectral responsivity. The TM-spectral responsivity of SABLG is enhanced by around twofold than the bare device. The simulation methodology and analytical analysis presented herein provide a versatile route for designing optimized polarimetric structures integrated into infrared imaging devices, offering superior capabilities to resolve linear polarization signatures.

## Introduction

The infrared (IR) imaging systems that sense and process complex information encoded in the incident optical wavefront are essential tools used in various applications, including those of medical^1,2^, military^3,4^, and environmental interest^5,6^. In these systems, the exploitation of IR polarization helps to distinguish the polarization state of emitted or reflected light^7^, suppressing complex backgrounds without thermal contrast^8^, thus aiding in target detection.

Various single-layer one-dimensional (1D) gratings, acting as wire grid polarizers, have emerged as tools for customizing the polarimetric properties through metal deposition-based patterning have been reported, such as elliptical Si nanowires^9,10^, nanorods^11^, metallic grating^12,13^, and liquid–crystal guest-host^14,15^. These nanostructures provide polarization over a wide range of wavelengths, but their structural limitations restrict the enhancements of polarization properties. Remarkably, optical nanostructures based on metal-dielectric composites, characterized by a dielectric separation layer and a nanoscale gap between upper and lower metal arrays, introduce the prospect of significantly extending functionality. Enriching performance further, integrating multiple layers of subwavelength 1D gratings engenders remarkable optical efficiency.

InAs/GaSb Type II Superlattice (T2SL) on GaSb substrate has emerged as a potential photodetector material, offering high performance by exceptional quantum efficiency and low dark current levels, which enables state-of-art infrared detection by controlling the band gap of an InAs/GaSb T2SL^16,17^. Coupling a detector with resonant structures such as Fabry–Perot^18,19^ and surface plasmon modes^20^ offer both resonant and broadband enhancement, which enables the encoding of spectral and polarization information, significantly enhancing the detector's signal-to-noise characteristics^21–26^.

Here, we investigate performance polarization properties (i.e., high transverse magnetic (TM) transmission, low transverse electric (TE) transmission, and high extinction ratio) of a self-aligned bilayer linear grating (SABLG). Our investigation delves into the effect of structural designs on the polarization properties of single-/double-layer linear gratings. Employing a multi-layer model grounded in the transfer matrix method, we unveil the potential to enhance both TM transmission and the extinction ratio of SABLG by optimizing geometrical dimensions and lateral positions of the upper linear gratings. The simulational result with MWIR InAs/GaSb T2SL photodetector also validates that the SABLG produces a pronouncedly improved TM–spectral responsivity and extinction ratio, assessed through TM- and TE-polarizations. The InAs/GaSb T2SL and SABLG structures could be manufactured using nanoassembly that circumvents undesirable fabrication issues (i.e., misalignments and cracks) in refs^27,28^.

## Results

**Figure 1:**
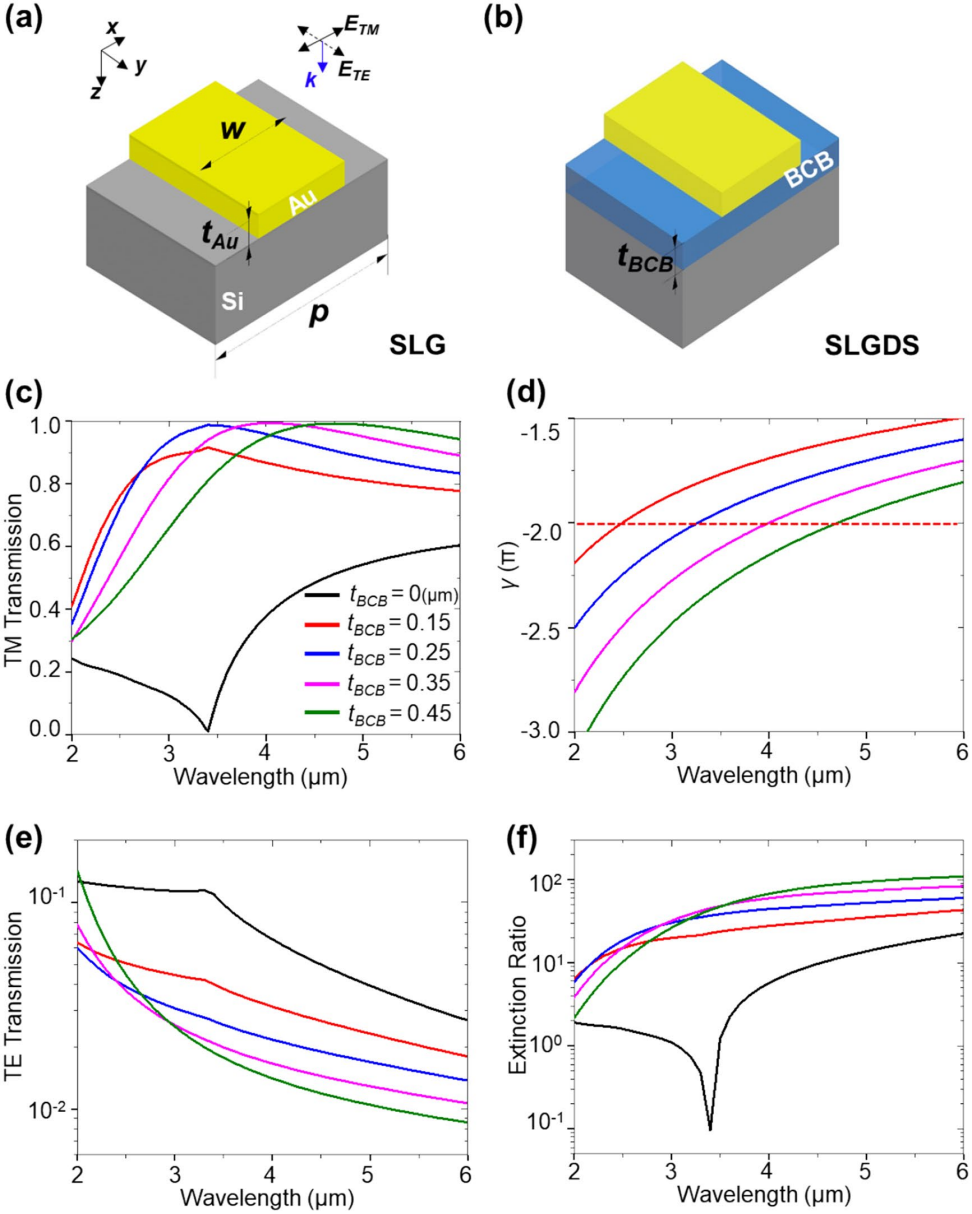
Schematic illustration of (**a**) single linear grating (SLG) polarizer and (**b**) single linear grating dielectric spacer (SLGDS) polarizer, where the grating period is 1 μm, the width of Au linear grating is 0.5 μm, the Au linear grating thickness is 0.1 μm and the BCB spacer thickness vary 0.15 μm to 0.45 μm. Simulation results of (**c**) TM-polarized transmission, (**d**) Fabry–Perot round-trip phase (γ), (**e**) TE-polarized transmission and (**f**) extinction ratio.

The transmission spectra of a single linear grating (SLG) and a single linear grating with a dielectric spacer (SLGDS) were simulated using the finite element method (FEM) in the wavelength range of 2–6 μm. Figure 1a illustrates an SLG configuration consisting of a gold (Au) grating on a silicon substrate where Au linear grating reflects TE waves parallel to the grating^29^. In this configuration, surface plasmon polariton resonance (SPPR) occurs at the interface between the silicon (Si) substrate and the Au linear grating, resulting in distinct transmission dips in the transmission spectra of TM waves^30,31^.

To mitigate unwanted SPPR, a dielectric spacer with a lower refractive index, here, Benzocyclobutene (BCB) with a frequency-independent refractive index of 1.54 and low extinction coefficient (*k*) in the MWIR range^32^, was employed as shown in Fig. 1b. In both SLG and SLGDS configurations, the grating period (*p*), the thickness of Au (*t*_*Au*_), the width of Au (*w*), and the thickness of BCB (*t*_*BCB*_) are 1 μm, 0.1 μm, 0.5 μm, and 0.15 to 0.45 μm, respectively. Figure 1c presents TM transmission spectra, where the dip at 3.4 μm wavelength shifts to a shorter wavelength and achieves wide bandwidth through the MWIR range by incorporating the BCB spacer. It generates an optical cavity, leading to Fabry–Perot resonance that enhances TM transmission for the SLGDS configuration. As a result, at 4 μm wavelength, TM transmission of SLGDS for each *t*_*BCB*_ is 2.3, 2.5, 2.6, and 2.5-fold higher than SLG (*t*_*BCB*_ = 0 μm), respectively.

A multiple-layer model based on a transfer matrix method was employed to study the wave propagation mechanism within the SLGDS structure, as depicted in Supplementary Fig. 1a. This model considers the reflection and transmission of the wave between the lower Au linear grating and Si substrates. The transfer matrix method was utilized to determine the overall reflection and transmission coefficients^33,34^, as described in the Methods section. Supplementary Fig. 1b demonstrates the calculated TM transmission spectra obtained using the multiple-layer model, showing consistent agreements with the numerical simulation of the SLGDS.

Figure 1d displays the calculated round-trip propagation phase (*γ*), revealing that the resonance condition shifts towards longer wavelengths as the *t*_*BCB*_ increases. The observed shift in resonance is crucial for achieving a wideband condition in the MWIR range TM transmission, highlighting the significance of optimizing *t*_*BCB*_ to enable wideband TM transmission. This condition occurs when the *γ* becomes an integer multiple of 2π. The phases, $\phi$(*r*_21_) and $\phi$(*r*_23_), at the interfaces of the BCB layer remain unaffected by variations in *t*_*BCB*_ (Supplementary Fig. 1c, d). With increasing *t*_*BCB*_, the wave propagation is longer within the BCB layer, causing the increased propagating phase factor (*β*) (Supplementary Fig. 1e), showing that the *β* is predominant in the Fabry–Perot resonance condition of SLGDS.

Figure 1e shows that the TE transmission of the SLGDS configuration surpasses that of the SLG configuration by at least 1.5-fold and threefold for *t*_*BCB*_ of 0.15 μm and 0.45 μm, respectively. As the *t*_*BCB*_ increases, the reflectance of TE waves rises above a wavelength of approximately 3 μm (Supplementary Fig. 2a). To visually demonstrate the impact of *t*_*BCB*_ on the SLGDS configuration, Supplementary Fig. 2b-e presents the electric field strength (|*E*|) at a wavelength of 4 μm. When *t*_*BCB*_ is 0.15 μm, |*E*| predominantly affects the region beneath the BCB spacer, resulting in higher TE transmission than a thicker BCB spacer. Figure 1f demonstrates that the extinction ratio of the SLGDS configuration, at *t*_*BCB*_ of 0.15 μm and 0.45 μm, exhibits enhancements of at least twofold and fivefold, respectively, while successfully preventing the occurrence of the undesired SPPR around ~ 3.4 μm.

**Figure 2:**
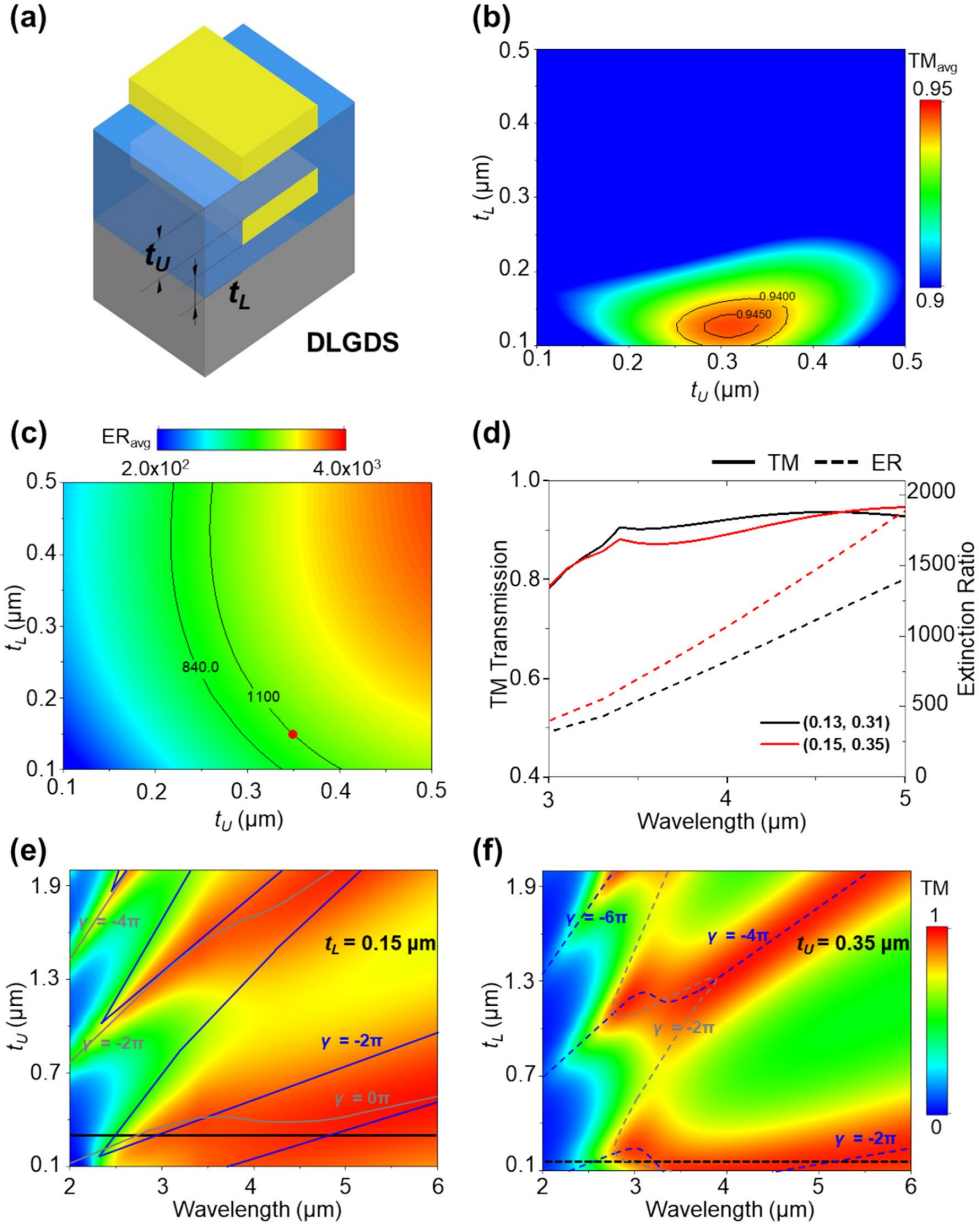
Schematic illustration of (**a**) double linear grating dielectric spacer (DLGDS) polarizer where *t*_*L*_ is thickness of between Si substrate and lower Au linear grating and *t*_*U*_ is thickness of between lower Au linear grating and upper Au linear grating. Colormap of average of (**b**) TM transmission and (**c**) extinction ratio at 3, 3.5, 4, 4.5 and 5 μm wavelengths as a function of *t*_*L*_ and *t*_*U*_. (**d**) TM transmission and extinction ratio of (0.13, 0.31) μm and (0.15, 0.35) μm (**e**,**f**) TM transmission varied with the thickness of *t*_*U*_ and *t*_*L*_ for the *t*_*L*_ = 0.15 μm and *t*_*U*_ = 0.35 μm(black line and dashed line). The blue lines representing round-trip phase(*γ*) based on the Air/lower Au linear grating-lower BCB and lower BCB/Si multiple-layer model indicate where Fabry–Perot resonance condition occur. The grey lines representing *γ* based on the Air/upper Au linear grating-upper BCB and upper BCB-lower Au linear grating/lower BCB-Si multiple-layer model indicate where Fabry–Perot resonance condition occur.

The double-layer linear grating dielectric spacer (DLGDS) polarizer, illustrated in Fig. 2a, was designed to enhance polarimetric function in the MWIR range. The schematic illustration depicts the thickness of the lower and upper dielectric spacer, denoted as *t*_*L*_ and *t*_*U*_. The lower dielectric spacer is positioned between the Si substrate and Au linear grating, while the upper dielectric spacer is placed between Au linear grating.

Figure 2b–f presents the computational results in achieving high TM transmission, wide bandwidth, and flat top response in the MWIR range to select an appropriate lower and upper dielectric spacer thickness. Figure 2b, c show colormaps of the average TM transmission (TM_avg_) and extinction ratio (ER_avg_) at wavelengths form 3, 3.5, 4, 4.5, and 5 μm as a function of *t*_*L*_ and *t*_*U*_. The results represent the maximum TM_avg_ of 0.946 and ER_avg_ of 839.8 at *t*_L_ = 0.13 μm and *t*_U_ = 0.31 μm, as marked (*t*_L_, *t*_U_) = (0.13, 0.31), which is approximately 1.1 and 30 times greater than the SLGDS configuration's TM_avg_ and ER_avg_ when *t*_*BCB*_ is 0.15 μm, respectively. Detailed results for this structure at each wavelength appear in Supplementary Figs. 3 and 4. The ER_avg_ exhibits increased value when both the *t*_*L*_ and *t*_*U*_ are increased, as shown in Fig. 2c. At (0.15, 0.35) (red dot), the TM_avg_ reaches 0.942, which is slightly below the maximum TM_avg_ value, while this combination value yields ER_avg_ of 1102.7, which is higher than the black contour line representing ~ ER_avg_ of (0.13, 0.31) in Fig. 2c.

Figure 2d compares the TM transmission and extinction ratio for the selected thickness combinations: (0.13, 0.31) and (0.15, 0.35). Both combinations ensure broadband and the flat top of TM transmission, while the extinction ratio of (0.15, 0.35) is 57%, 109%, and 160% larger than (0.13, 0.31) at the wavelength of 3, 4, 5 μm, indicating a superior polarizer.

The colormaps depicted in Fig. 2e, f illustrate the variation in the TM transmission as the *t*_*U*_ and *t*_*L*_ are varied from 0.1 to 2.0 μm while fixing when *t*_*L*_ = 0.15 and *t*_*U*_ = 0.35 μm, respectively. The blue lines, derived from the Air/lower Au linear grating-lower BCB and lower BCB/Si model(Supplementary Fig. 5a), and the grey lines, based on the Air/upper Au linear grating-upper BCB and upper BCB-lower Au linear grating/lower BCB-Si model (Supplementary Fig. 5b), are calculated *γ* that satisfy Fabry–Perot resonance condition, which indicates where the high TM transmission occurs. The variation in *t*_*U*_ and *t*_*L*_ leads to a phase shift denoted by $\phi$(*r*_21_) for *t*_*U*_ and $\phi$(*r*_23_) for *t*_*L*_ due to double Au linear grating and BCB spacer, generating multiple Fabry–Perot resonances. Also, the alteration in *t*_*U*_ and *t*_*L*_ increased the *β,* further affecting multiple Fabry–Perot resonances. Therefore, the DLGDS configuration's *t*_*U*_ and *t*_*L*_ values are crucial to obtaining flat and high TM transmission enhanced by multiple Fabry–Perot resonances. The solid black line and black dash line in these figures indicate that the wideband TM transmission that covers the entire MWIR wavelength ranges from 3 to 5 μm when *t*_*U*_ of 0.35 μm and *t*_*L*_ of 0.15 μm, respectively. The detailed TE and ER results are represented in Supplementary Fig. 6. With optimal values of *t*_*U*_ and *t*_*L*_ for achieving high and wide TM transmission, the structure can be used as a broadband polarizer with high transmission efficiency and extinction ratio.

**Figure 3:**
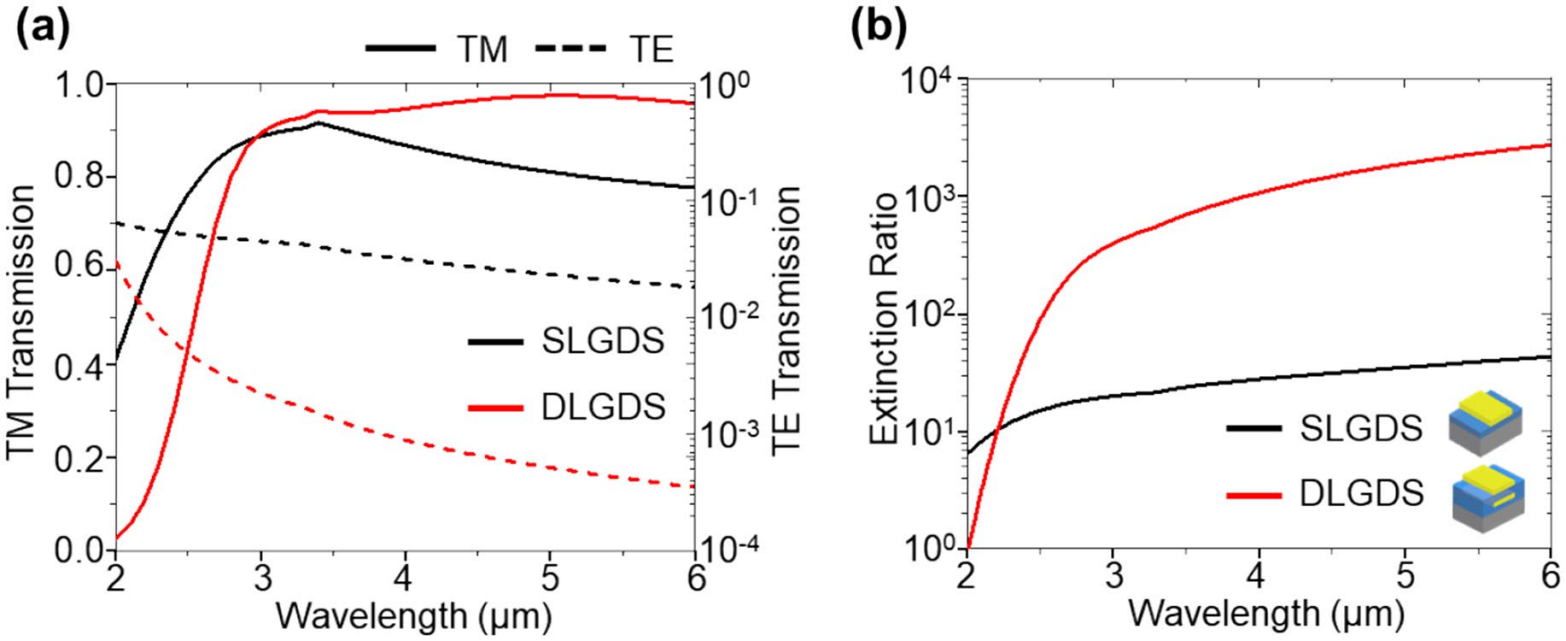
Simulation results of (**a**) TM and TE transmission and (**b**) extinction ratio of DLGDS of inserted illustrations.

Figure 3a, b show the simulated transmission spectra and extinction ratio for SLGDS (in black) and DLGDS (in red) when *t*_*U*_ = 0.35 μm and *t*_*L*_ = 0.15 μm. The TM transmission of SLGDS decreases monotonously in the MWIR region of 3.4 – 6 μm, while that of DLGDS remains barely changed over 0.9, resulting in an almost flat top TM transmission. It indicates that DLGDS achieves 1.2 times higher TM transmission than SLGDS at 5 μm wavelength and also shows higher TM transmission across the entire wavelength region of interest compared to the SLGDS. The improved performance of DLGDS is attributed to the Au linear grating with the dielectric spacer on SLGDS, which leads to the formation of constructive reflection in the BCB optical cavity between the upper and lower Au linear gratings. TE transmission of the DLGDS is significantly lower than that of SLGDS due to the sequential exposure of Au linear grating, attributable to the reflection of the parallel oscillating electric field of the TE wave on the Au linear grating in the double-layer structure^29,32^. Consequently, the DLGDS exhibits an over ~ 40-fold than SLGDS extinction ratio, the TM to the TE transmission ratio serving as a performance indicator of the MWIR polarizer, as indicated in Fig. 3b.

**Figure 4:**
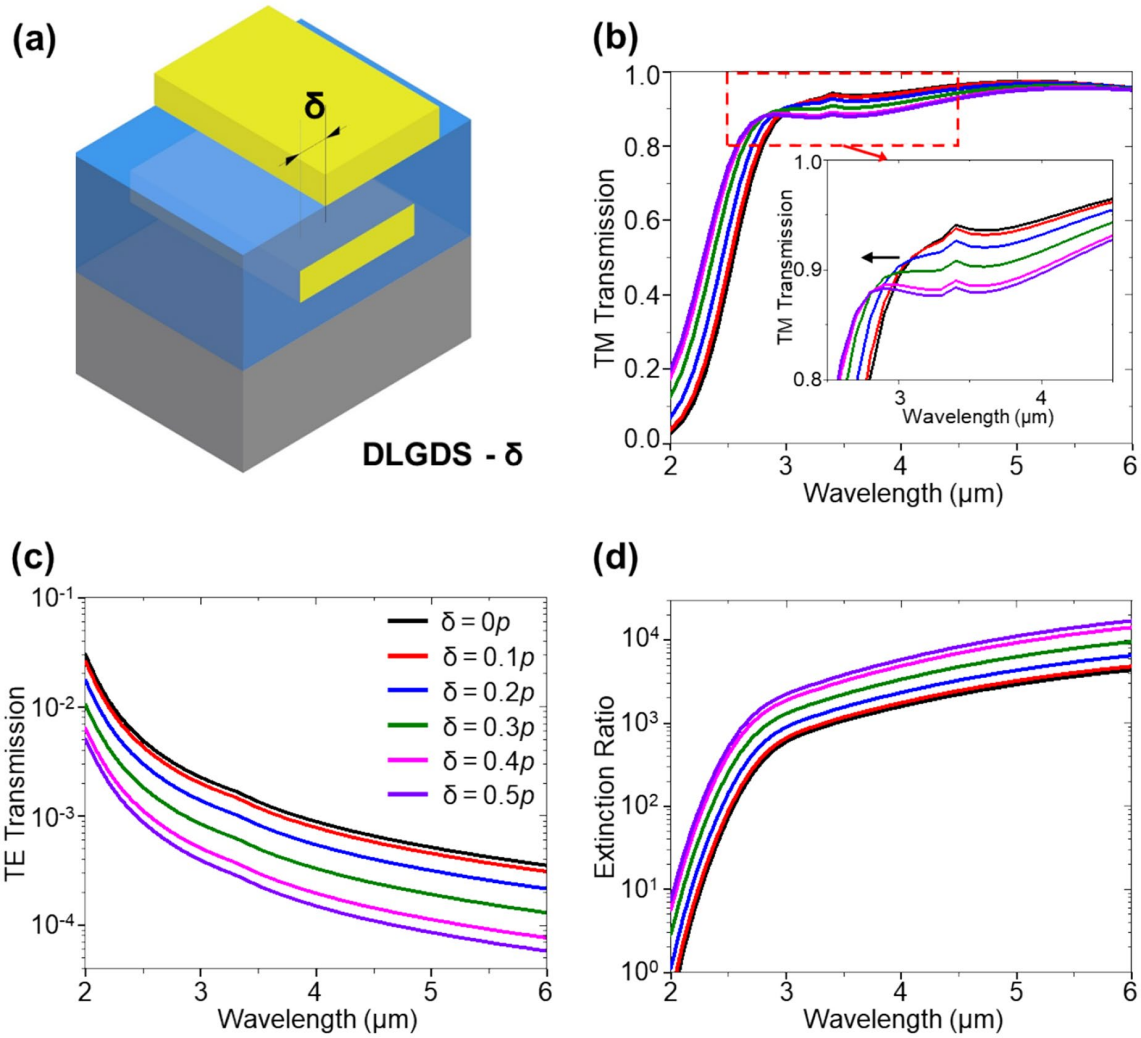
(**a**) Schematic illustration of DLGDS according to lateral shifting (δ) upper Au linear grating. Simulation results of (**b**) TM transmission, (**c**) TE transmission and (**d**) extinction ratio.

Figure 4a shows the schematic illustration of DLGDS – lateral shift (δ) (ranging from 0 p to 0.5*p*), where δ represents the distance by which the upper linear grating is shifted to enhance the exposure of the TE wave to the Au linear grating. The corresponding schematic illustrations for varied δ are summarized in Supplementary Fig. 7a–e. As δ shifts from 0 to 0.5*p*, the TM transmission in Fig. 4b is reduced due to the increasing exposure area of lower Au linear gratings to the incident light, causing an enhancement in reflection (Supplementary Fig. 7f). Moreover, the TM transmission exhibits a blue-shifted caused by a shift in the wavelength of the Fabry–Perot resonance at a wavelength of ~ 3 μm, illustrated in Supplementary Fig. 7g. At δ = 0.5*p*, the upper and lower Au linear gratings are perfectly misaligned, resulting in the maximum exposure of the TE-polarized light to the Au linear gratings (Fig. 4c), which leads to the higher extinction ratio in Fig. 4d. This shows that the extinction ratio is dominated by TE transmission in the relation between TM transmission/TE transmission.

**Figure 5:**
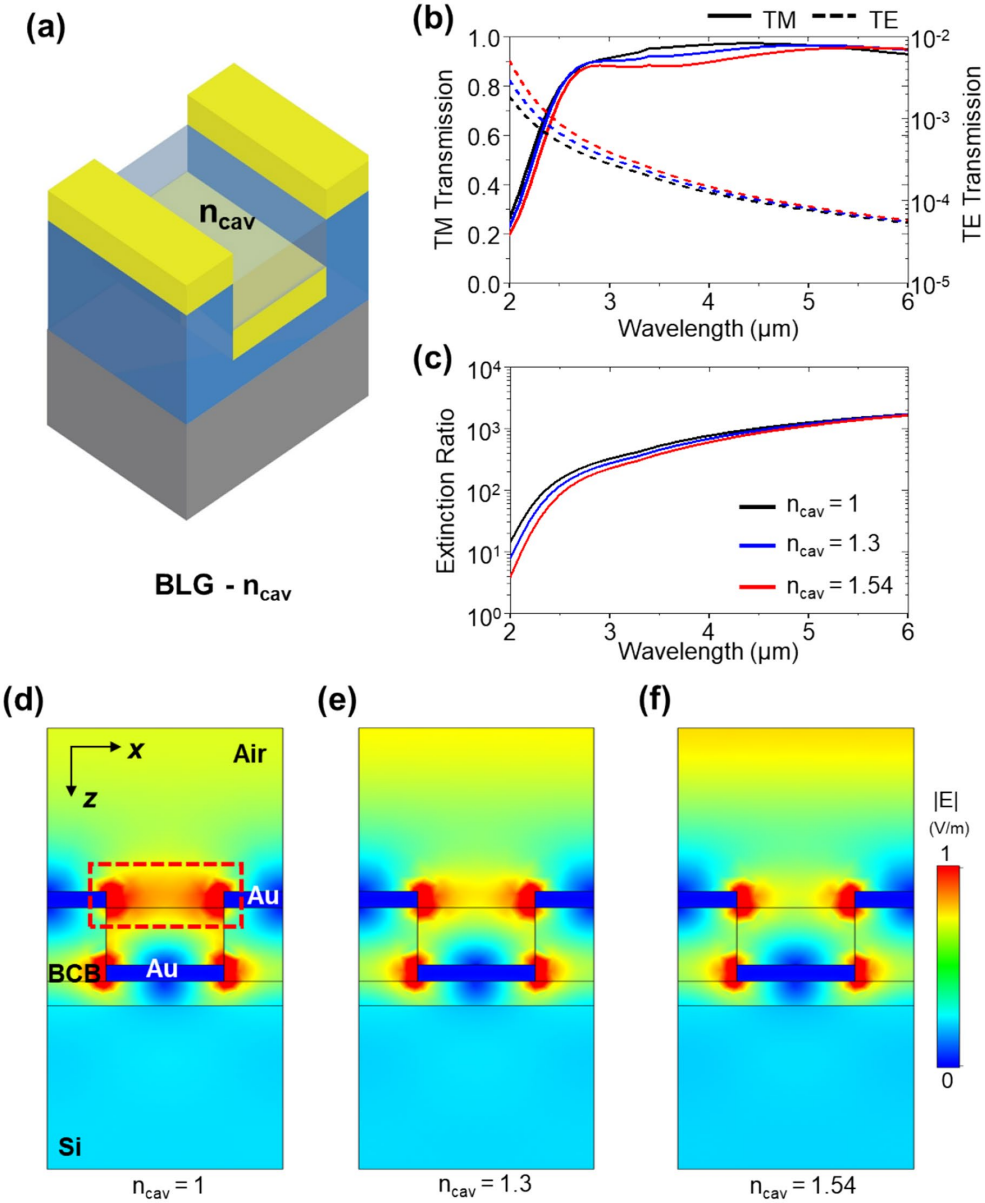
Schematic illustration of (**a**) bilayer linear grating polarizer (BLG—n_cav_) filled with spacer which has refractive index, n_cav_. (**b**) TM transmission and reflection and (**c**) TE transmission and extinction ratio. Simulated electric field distributions for (**d**) n_cav_ = 1, (**e**) n_cav_ = 1.3, (**f**) n_cav_ = 1.54.

Figure 5a shows the bilayer linear grating configuration (BLG—n_cav_) for DWGDS when δ = 0.5*p*, which has been investigated as the best IR polarizer in the preceding analysis. The investigation focuses on the effect of the refractive index of the top dielectric layer on this structure, where the layer is exposed to the air. The refractive index of the top dielectric layer varies from the refractive index of BCB (n_BCB_ = 1.54) to air (n_air_ = 1). In Fig. 5b, when n_cav_ is reduced from 1.54 to 1, TM transmission increases in the MWIR range while TE transmission decreases, resulting in an enhanced extinction ratio (Fig. 5c). In the TM-polarized light, |*E*| at 4 μm wavelength shows enhanced |*E*| at the edge of upper Au linear grating between the top dielectric layer as the n_cav_ decreased, illustrated in Fig. 5d–f. This strong enhanced electric confinement at the edge of the upper Au linear grating increases TM transmission at n_cav_ = 1. It exhibits high TM transmission and lower TE transmission, resulting in an enhanced extinction ratio compared to DLGDS and DWGDS—δ:0.5*p*, as shown in Supplementary Fig. 8a, b. This configuration enables nanotransfer printing or nanoimprinting technologies, providing only one metal deposition step, thereby denoting self-aligned bilayer linear grating(SABLG).

This study investigates the spectral responsivity of integrating various designed structures (SLG, SLGDS, DLGDS, and SABLG) into MWIR InAs/GaSb T2SL photodetector. The overall polarizer performance of the T2SL device across the target wavelength spectrum (2.5–5.5 µm) was simulated using a finite element method, and details of device simulation are illustrated in the Methods section.

**Figure 6:**
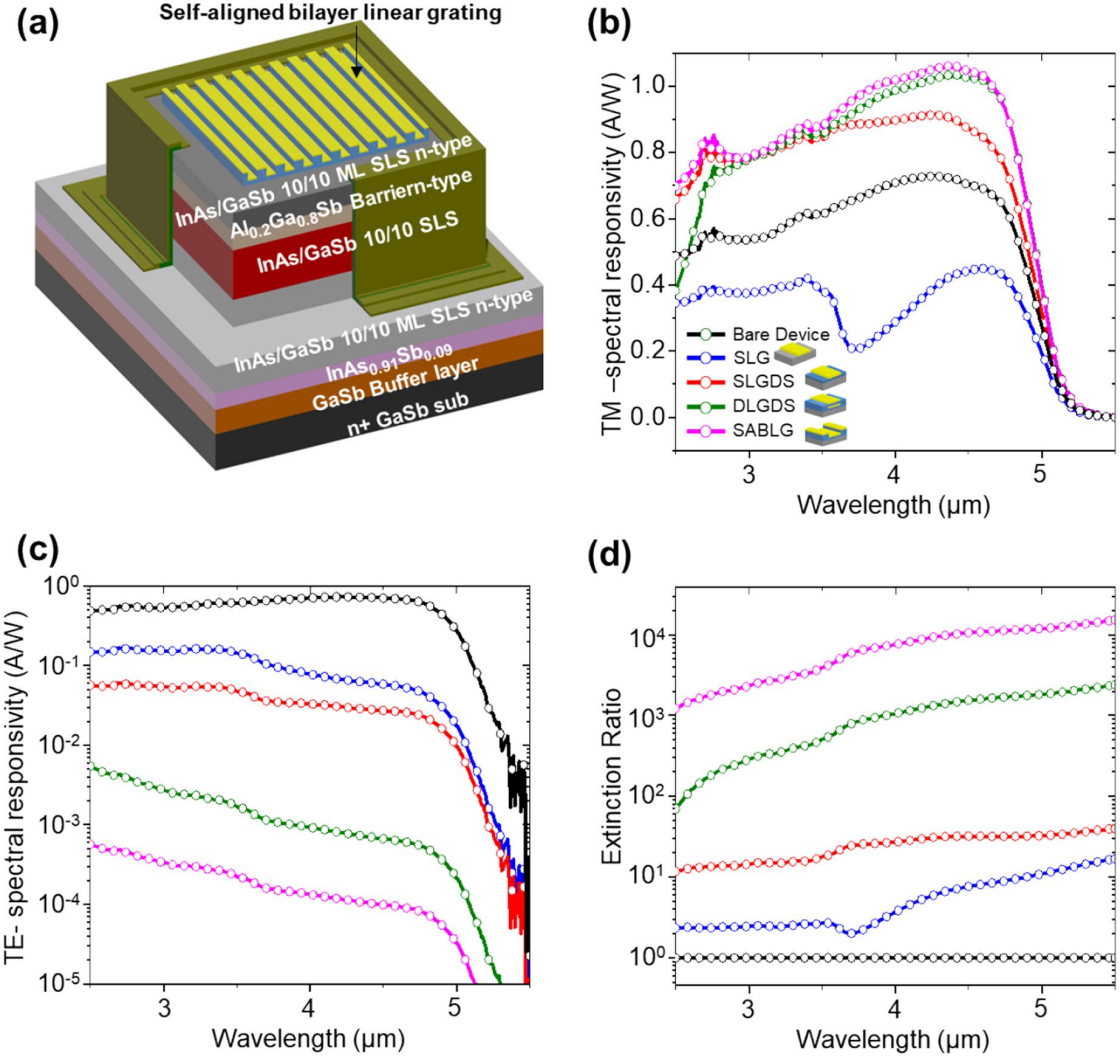
(**a**) Schematic illustration of polarizer integrated MWIR InAs/GaSb T2SL photodetector. Simulation results of (**b**) TM-spectral responsivity, (**c**) TE-spectral responsivity and (**d**) spectral response polarizability.

Figure 6a depicts the SABLG structures integrated into the InAs/GaSb-based T2SL device. The device includes a 300 nm GaSb buffer layer and a 200 nm thick InAs0.91Sb0.09 acting as an etch stop layer on an n + GaSb substrate. The bottom contact layer consists of n-type InAs/Gasb (80 periods), forming each of 10 monolayers (ML), and the active layer is designed as strained layer superlattices (SLS) containing 10 ML of InAs/10 ML of GaSb SLS (300 periods). 100 nm thick n-Al0.2Ga0.8Sb heterostructure barrier is grown to complete the device structure. Finally, n-type 10 ML InAs/10 ML GaSb SLS (30 periods) are grown as a top contact layer, with the designed configuration subsequently placed atop the last T2SL device. Supplementary Fig. 10 shows that the experimental measurement at 77 K, bias voltage − 1 V (black lines), is in excellent agreement with simulated responsivity (blue lines).

The SABLG structure is integrated into the top of the device. (Supplementary Fig. 9a–d for a bare device, SLG, SLGDS, and DLGDS-integrated configurations with identical dimensions; *p* (1 μm) and *t*_*Au*_ (0.1 μm), respectively). Figure 6b–d shows the TM-, TE-spectral responsivity, and extinction ratio as a function of the operating wavelength for the integrated structures (SLGDS, DLGDS, and SABLG) with the MWIR InAs/GaSb T2SL photodetector. In Fig. 6b, the TM-spectral responsivity of the SABLG-integrated device achieves an improved value of ~ 1.48 A/W at a peak wavelength of about 4.5 μm, surpassing the peak values of both the bare (~ 0.7A/W) and SLG-integrated devices (~ 0.62 A/W). The increased responsivity for SABLG-integrated devices indicates stronger Fabry–Perot resonance presenting low reflection and improving absorptance in the active layer in Supplementary Fig. 11a, b, while that of the SLG (represented in a dotted blue line) exhibits a lower spectral responsivity than the bare T2SL device, attributed to impedance mismatching and dip caused by SPPR at 3.4 μm. The x-and z-component electric field distribution in a plane parallel to the xz-plane within the active layer (${E}_{Act}^{x}$, ${E}_{Act}^{z}$) of the devices at 4 μm wavelength at the center of the device ($x=0$) along z (0 $\mathrm{\mu m} \le$ z $<$ 2 μm) in Supplementary Fig. 11c. shows that ${E}_{Act}^{x}$ and ${E}_{Act}^{z}$ of the SABLG integrated device are enhanced compared to the bare device, which presents the responsivity enhancement (Supplementary Fig. 11d, e).

In Fig. 6c, a dotted black line presents the TE-spectral responsivity of the bare device, which is identical to TM-spectral responsivity (Fig. 6b), indicating the absence of polarization. In contrast, the SLG, SLGDS, DLGDS, and SABLG-integrated device exhibits an apparent polarization effect. Notably, the SABLG-integrated device's responsivity decreases by over ~ 5286-fold compared to the bare device.

As indicated in Fig. 6d, the SABLG structure offers an average of ~ 2 times higher spectral responsivity than the bare device in the 2.5–5.5 μm wavelength range, highlighting its superior polarization performance compared to the other structure designs. The results conclusively show that the integration of the SABLG structure enables deterministic control of the waveguide resonance in the T2SL photodetector, primarily due to the presence of Fabry–Perot resonances. This behavior confirms the success of the SABLG structure in achieving improved polarization performance and validates its potential for advanced applications in MWIR polarimetric systems.

## Discussion

The results outlined herein illustrate the design of linear grating structures capable of achieving wideband and uniform high transmission, which significantly enhances the extinction ratio in the MWIR range. The SABLG architecture brings TM transmission enhancement and uniformity attributed to the Fabry–Perot resonance in dielectric spacers and the optimal height of upper and lower dielectric spacers. Integrating the SABLG into MWIR InAs/GaSb T2SL photodetectors substantially enhances TM-spectral responsivity while reducing TE-spectral responsivity, thereby improving the extinction ratio. These findings highlight the promising potential of the SABLG structure, which can be applied effectively to sense and parse information stored in the light emerging from a target and process it in the analog domain at the speed of light, step forward to the next generation of infrared detectors.

## Methods

### Analytical calculation for Fabry–Perot resonance

The transfer matrix method can describe the underlying mechanism for high transmission polarizer configurations using a multiple–layer model^33,34^. The following equation can calculate the overall transfer matrix (*M*)

1 $$M= {M}_{upper}\cdot {M}_{BCB}\cdot {M}_{lower}$$

*M*_*upper*_, *M*_*BCB*,_ and *M*_*lower*_ are the transfer matrices for the air-Au linear grating-BCB(1), BCB(2) and BCB-Si(3) layers, respectively. The transfer matrixes can be expressed by complex coefficient *r*, *t*

2 $${M}_{upper}=\left(\begin{array}{cc}{{t}_{21}- r}_{12}{r}_{21}/{r}_{12}& {r}_{12}/{t}_{12}\\ -{r}_{21}/{t}_{12}& 1/{t}_{12}\end{array}\right)$$

3 $${M}_{BCB}=\left(\begin{array}{cc}{e}^{i\beta }& 0\\ 0& {e}^{-i\beta }\end{array}\right)$$

4 $${M}_{lower}=\left(\begin{array}{cc}{{t}_{21}- r}_{12}{r}_{21}/{r}_{12}& {r}_{12}/{t}_{12}\\ -{r}_{21}/{t}_{12}& 1/{t}_{12}\end{array}\right)$$

The parameters *t*_*ij*_ and *r*_*ij*,_ represent signal input in *i* layer and response to the input signal at *j* layer, denoting wave transmission *i* to *j* and incident wave *i* being reflected at *j* layer interface, respectively. Within the BCB layer, only propagating waves are present in transmitted light. The reflection and transmission coefficients *r*, *t* are as follows:

5 $$r=\frac{{r}_{12}+{\alpha r}_{23}{e}^{-2i\beta }}{1-{r}_{21}{r}_{23}{e}^{-2i\beta }}, t=\frac{{t}_{12}{t}_{23}{e}^{-i\beta }}{1-{r}_{21}{r}_{23}{e}^{-2i\beta }}$$

The parameters *r*_12_ and *r*_21_ are the reflection coefficients at the front and the back sides of the upper Au linear grating, respectively. *r*_23_ is the reflection coefficient from the lower Au linear grating. *β* is the propagating phase factor of *β* = n_BCB_⋅*k*⋅*t*_*BCB*_ in the BCB layer, where n_BCB_, *k*, and *t*_*BCB*_ are the refractive index of BCB, the wave vector, and the BCB thickness, respectively. *α* is expressed by

6 $$\alpha = {t}_{21}{t}_{12}-{r}_{21}{r}_{12}$$

where *t*_12_ and *t*_21_ are the forward and backward transmission coefficients through the upper Au linear grating, respectively. The entire transmission and reflection are calculated using *r* and *t* coefficients derived from (S5).

7 $${T}_{cal}= {|t|}^{2}, {R}_{cal}={|r|}^{2}$$

Here, to achieve Fabry–Perot resonance caused by multiple reflections on BCB cavity interfaces, the constructive interface condition within the BCB layer is followed by

8 $$\gamma =\phi \left({r}_{21}\right)+\phi \left({r}_{23}\right)-2\cdot \beta =2m\pi\, for\, \left|m\right|=\mathrm{0,1},2, \cdots$$

where *γ* is the round-trip propagation phase within the BCB layer, and $\phi$ is the phase of reflected wave at each layer's interface.

### Numerical simulation

The polarizer configurations are modeled with COMSOL Multiphysics using an RF module in Two dimensional (2D) model. The simulation is conducted to analyze the performance of the polarizer and its underlying mechanism. The incident port is placed on top of the model, and the receive port is placed on the bottom of the Si substrate. The transverse electric (TE) parallel to the linear grating direction is applied, and the wave direction of an electric field of incident light is set as the z-direction. In contrast, for the transverse magnetic (TM), the magnetic field of incident light is set as z-direction. The left and right boundaries are set as periodic boundary conditions. The refractive index of BCB and silicon (Si) is 1.54 and 3.4, respectively. The Drude model is applied to the gold (Au) with the plasma frequency of *w*_*p*_ = 1.38 × 10^16^ Hz and the collision frequency of *w*_*c*_ = 5.71 × 10^13^ Hz^35^.

### Device simulation

Supplementary Fig. 12 presents the schematic illustrations of a simplified T2SL device incorporating the polarizer configurations (SLG, SLGDS, DLGDS, SABLG) positioned on the ‘Top’ layer. In the COMSOL Multiphysics, a two-dimensional unit cell was modeled to generate a cross-section of the device using the Floquet periodic boundary condition. The incident plane wave propagates in the z-direction, and the electric field is polarized to the x-direction (y-direction), and the TM-polarized (TE-polarized) wave is applied.

The spectral responsivity of T2SL can be calculated using the following equation.

$$R\left(\lambda \right)=(\frac{{\eta }_{IQE}(\lambda )\cdot {N}_{0}(\lambda )\cdot {e}^{-}}{{P}_{BB}(\lambda ,{T}_{0})})\cdot \iint \frac{\omega \cdot {\varepsilon }^{"}\cdot \{{\sum }_{j=x,y,z}{|{E}_{Act}^{j}\left(\lambda \right)|}^{2}\}}{2}d{S}_{Act}$$

where the $${\eta }_{IQE}$$ is supposed to 1, $${N}_{0}(\lambda )$$ is the incident photon flux, $${P}_{BB}(\lambda ,{T}_{0})$$ is the power radiation by a blackbody of a given temperature ($${T}_{0})$$, $${\varepsilon }^{"}$$ is the imaginary permittivity of the active layer, and $${E}_{Act}^{j}\left(\lambda \right)$$ is the *j*-direction electric field of the active layer. For the bare device and the polarizer (//y) integrated device as shown in Supplementary Fig. 12, the equation can be applied respectively as following equations,

$${R}_{bare}\left(\lambda \right)\approx C\cdot \iint {\varepsilon }^{"}\cdot \left\{{\left|{E}_{Act}^{x}\left(\lambda \right)\right|}^{2}\right\} d{S}_{Act}$$

$${R}_{TE}\left(\lambda \right)\approx C\cdot \iint {\varepsilon }^{"}\cdot \left\{{\left|{E}_{Act}^{y}\left(\lambda \right)\right|}^{2}\right\} d{S}_{Act}$$

$${R}_{TM}\left(\lambda \right)\approx C\cdot \iint {\varepsilon }^{"}\cdot \left\{{\left|{E}_{Act}^{x}\left(\lambda \right)\right|}^{2}+{\left|{E}_{Act}^{z}\left(\lambda \right)\right|}^{2}\right\} d{S}_{Act}$$

where *C* = ${\pi \cdot c}_{0}\cdot {e}^{-}/1.24$ and *c*_0_ is the speed of light. There is no different spectral responsivity of bare device for the polarized state. However, for the polarizer integrated device in TE state incidence (//y), the electric field intensity of the x and z components can be ignored. In the case of TM state incidence (//x), the y component of electric field intensity can be neglected.

### Supplementary Information

Supplementary Figures.

## Data Availability

The data that support the findings of this study are available from the corresponding authors upon reasonable request.
