# Supplementary material for: Simulational investigation of self-aligned bilayer linear grating enabling highly enhanced responsivity of MWIR InAs/GaSb type-II superlattice (T2SL) photodetector
Source: Sci Rep. 2024 Jan 24;14:2050. doi: 10.1038/s41598-024-52113-4 (PMC10808251; doi:10.1038/s41598-024-52113-4)
Supplement: Supplementary file 1 — Supplementary Figures. [file 41598_2024_52113_MOESM1_ESM.pdf]

Supplementary Information for

Simulational Investigation of Simulational Investigation of Self-Aligned Bilayer Linear Grating Enabling Highly Enhanced Responsivity of MWIR InAs/GaSb Type-II Superlattice (T2SL) Photodetector

Minseok Lee[1†], Zahyun Ku[2†], Seungjin Jeong[1], Jehwan Hwang[3], Junghyun Lee[1,4], Junoh Kim[4], Sang-Woo Kang[4], Augustine Urbas[2], Hagyoul Bae[5*], Bongjoong Kim[1*]

[1]Department of Mechanical and System Design Engineering, Hongik University, Seoul 04066, Republic of Korea

[2]Materials and Manufacturing Directorate, Air Force Research Laboratory, Wright-Patterson Air Force Base 45433, United States

[3]Optical Lens Materials Research Center, Korea Photonics Technology Institute (KOPTI), Gwangju 61007, Republic of Korea

[4]Advanced Instrumentation Institute, Korea Research Institute of Standards and Science, Daejeon 34113, Republic of Korea

[5]Department of Electronic Engineering, Jeonbuk National University, Jeonju 54896, Republic of Korea

*Correspondence and requests for materials should be addressed to B.K. (email: bjkim23@hongik.ac.kr) or H.B. (email: hagyoul.bae@jbnu.ac.kr)

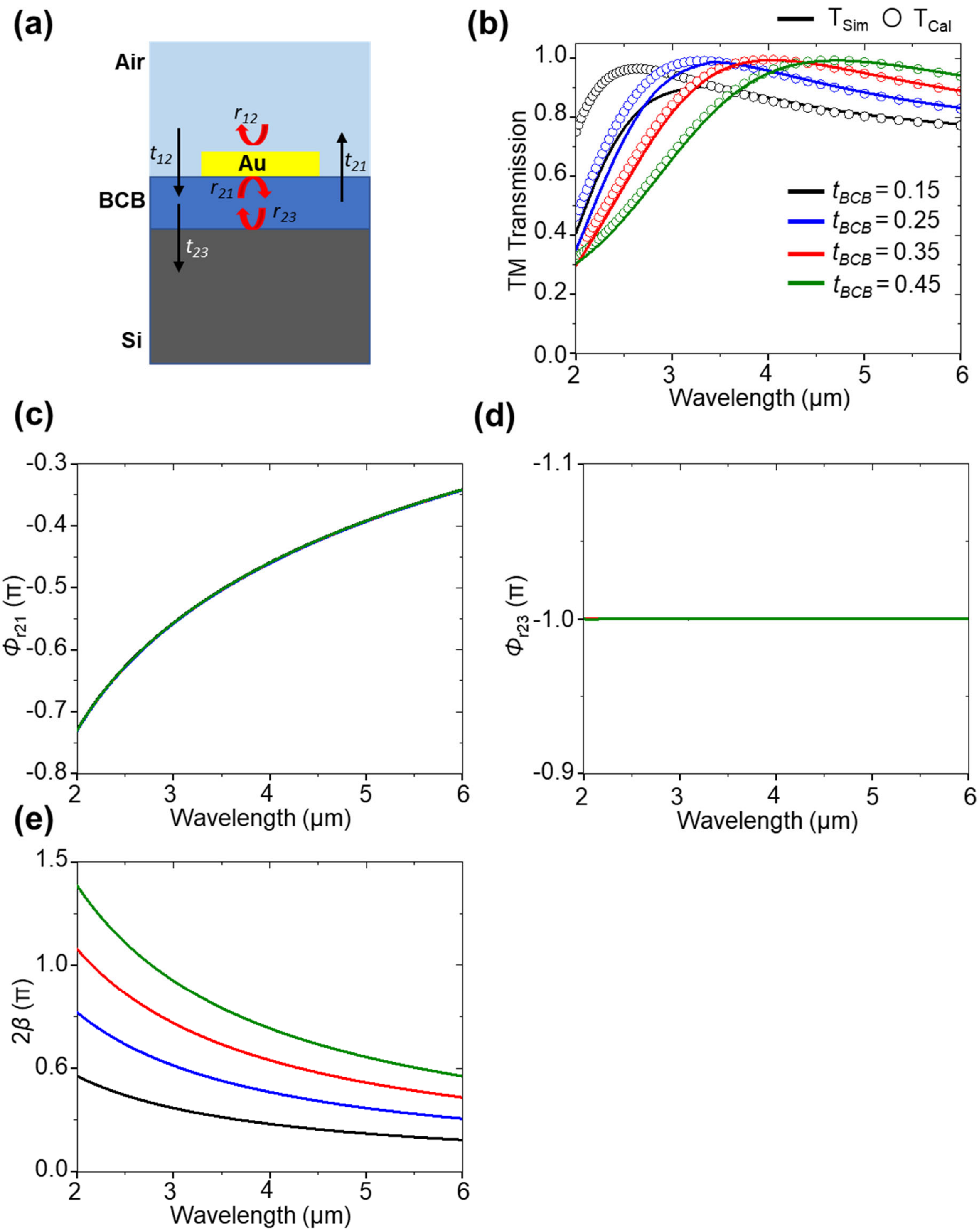

Supplementary figure S1 – (a) Schematic illustration of multiple-layer model using transfer matrix method, (b) TM transmission of simulation and calculation results, (c), (d), (e) calculated phase results for varying t_{BCB}.

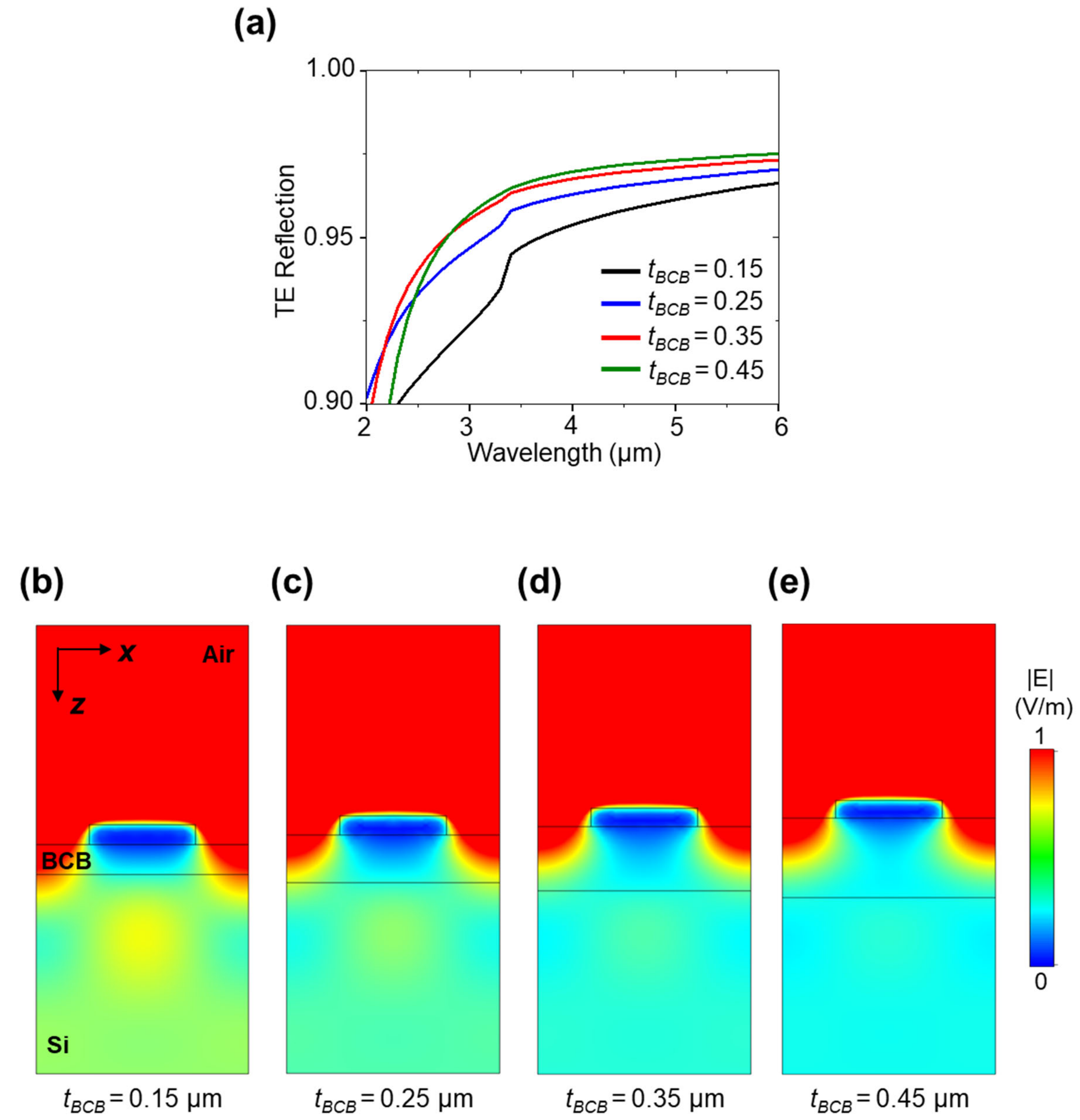

Supplementary figure S2 – (a) TE reflection for varying t_{BCB} from 0.15 µm to 0.45 µm, (b), (c), (d), (e) simulated electric field distribution.

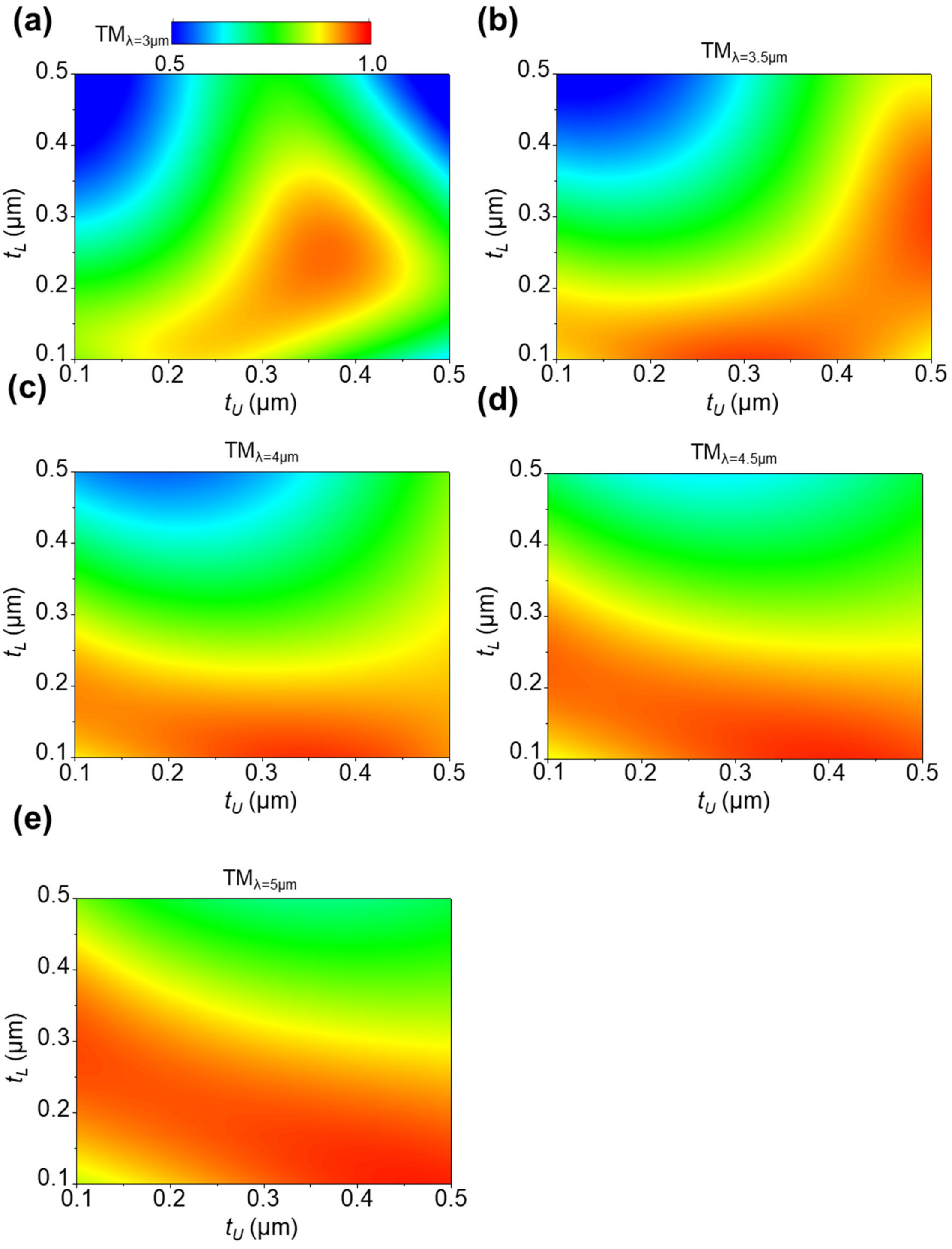

Supplementary figure S3 – Colormap of TM transmission at (a) 3 μm, (b) 3.5 μm (c) 4 μm, (d) 4.5 μm, (e) 5 μm as a function of t_L and t_U, respectively.

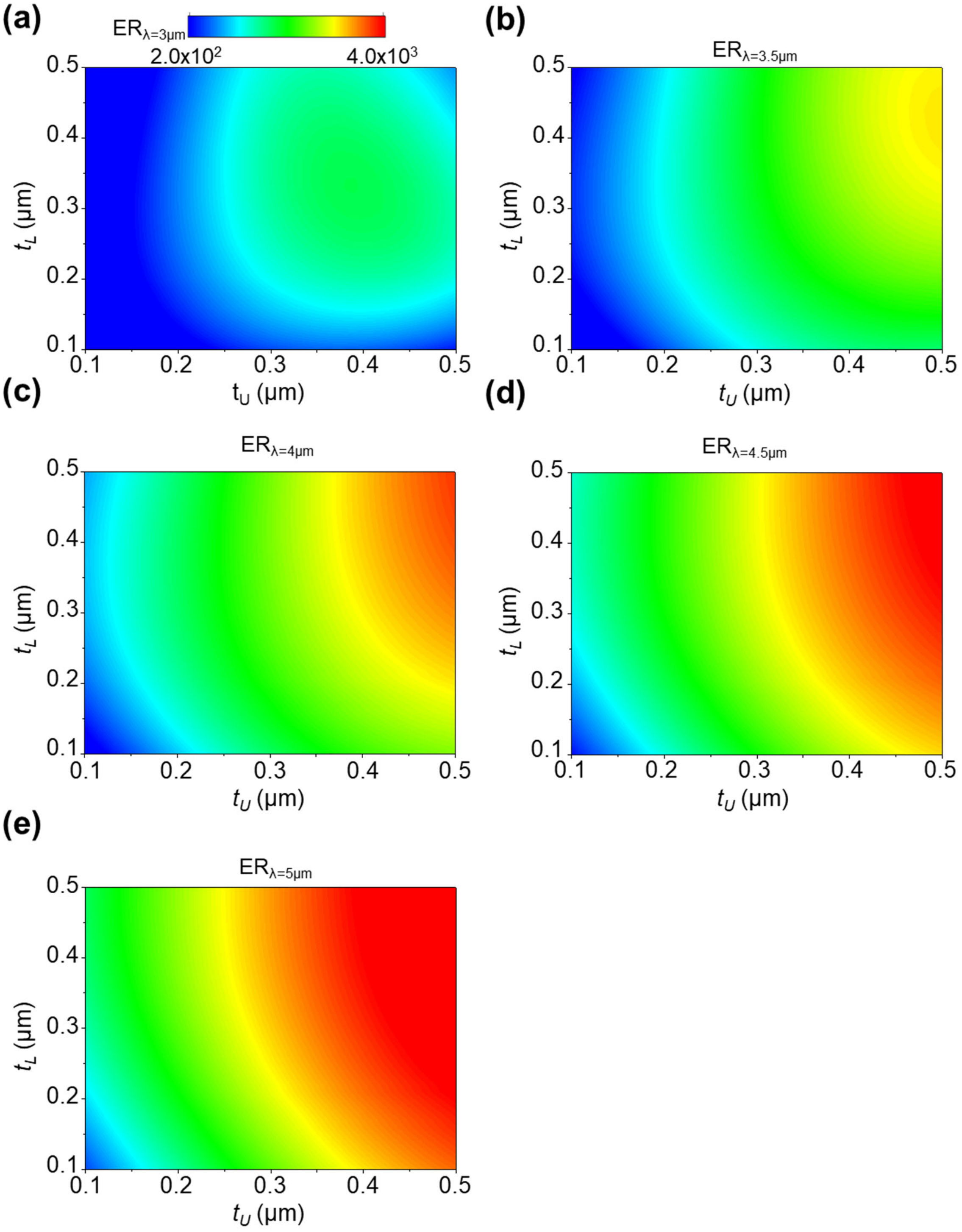

Supplementary figure S4 – Colormap of ER transmission at (a) 3 µm, (b) 3.5 µm (c) 4 µm, (d) 4.5 µm, (e) 5 µm as a function of t_L and t_U, respectively.

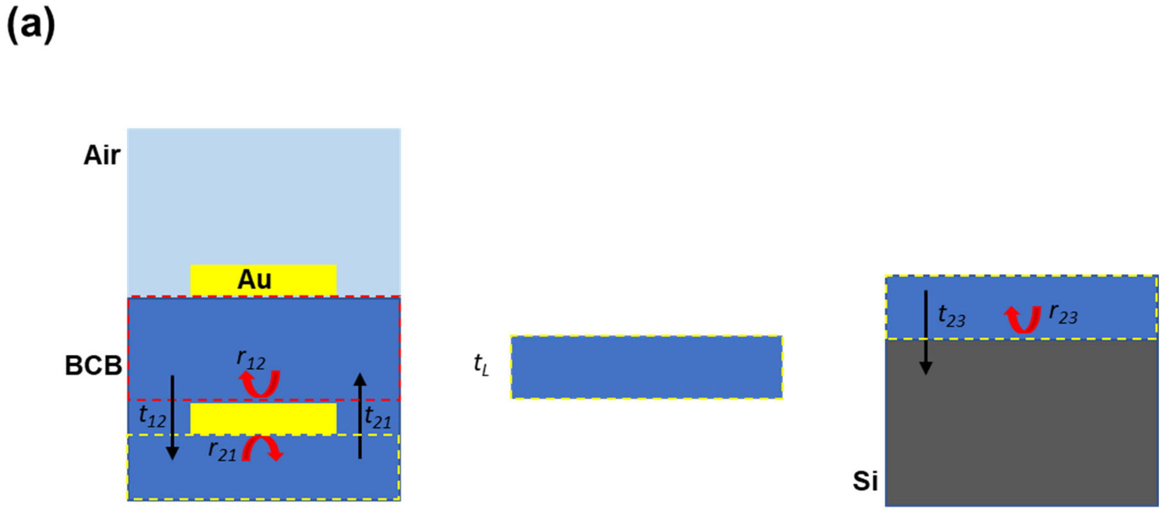

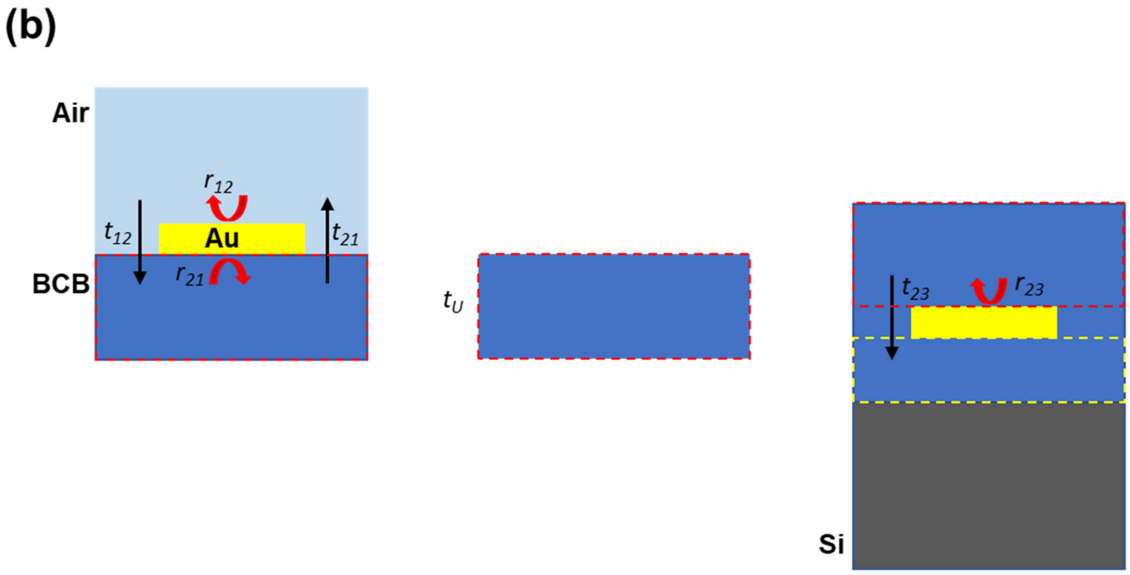

Supplementary figure S5 – Schematic illustration of multiple-layer model using transfer matrix method, (a) Air/lower Au linear grating-lower BCB (yellow dotted box) and lower BCB/Si, (b) Air/upper Au linear grating-upper BCB(red dotted box) and upper BCB-lower Au linear grating/lower BCB-Si model.

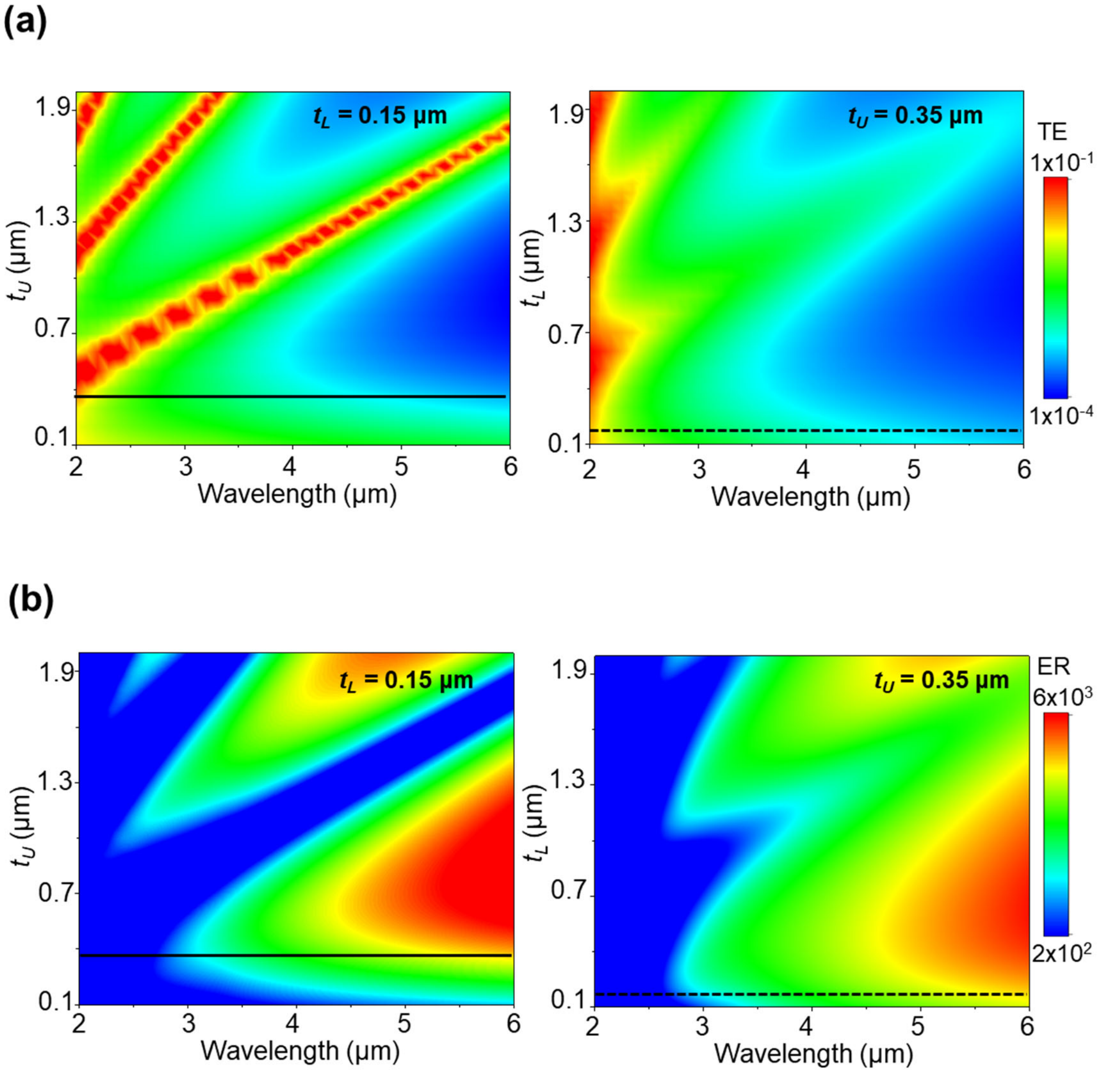

Supplementary figure S6 – Colormap of (a) TE transmission and (b) Extinction ratio varied with the thickness of t_U and t_L for the t_L = 0.15 μm and t_U = 0.35 μm.

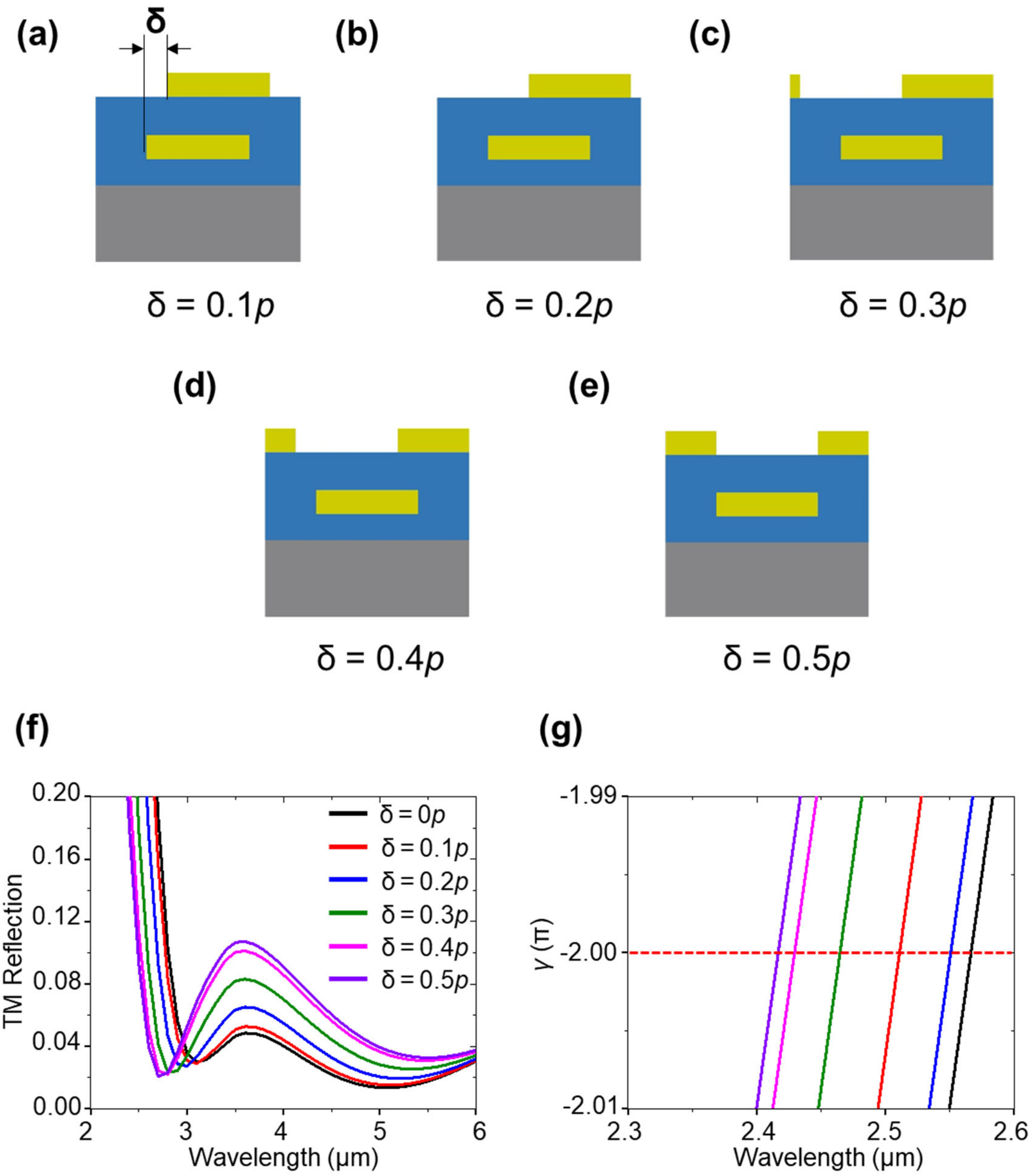

Supplementary figure S7 – (a)-(e) Schematic illustration of DLGDS's upper Au linear grating with lateral shift (δ) 0.1*p* to 0.5*p*, (f) TM reflection as upper Au linear grating shifted as δ, (g) changing of Fabry-Perot resonance condition wavelength as upper Au linear grating shifted as δ.

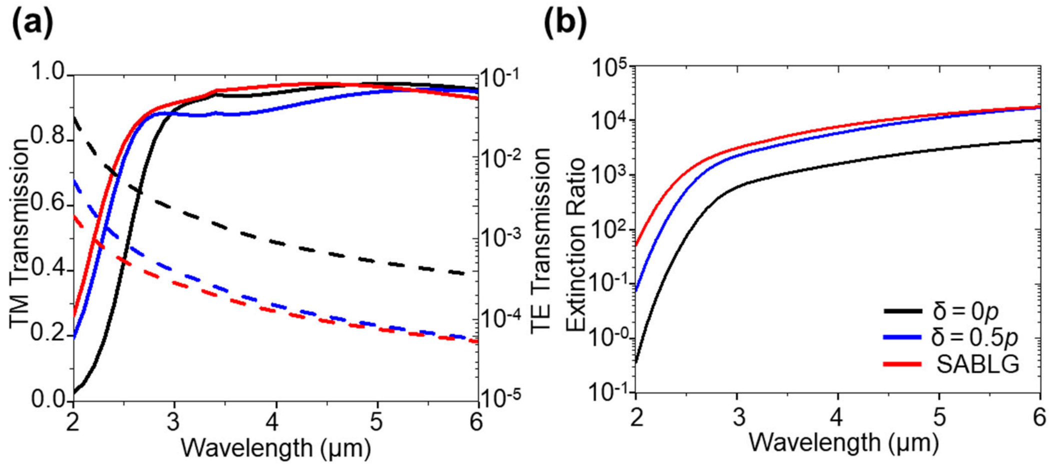

Supplementary figure S8 – (a) TM, TE transmission, (b) extinction ratio, and (c) schematic illustration of transfer printing fabrication method.

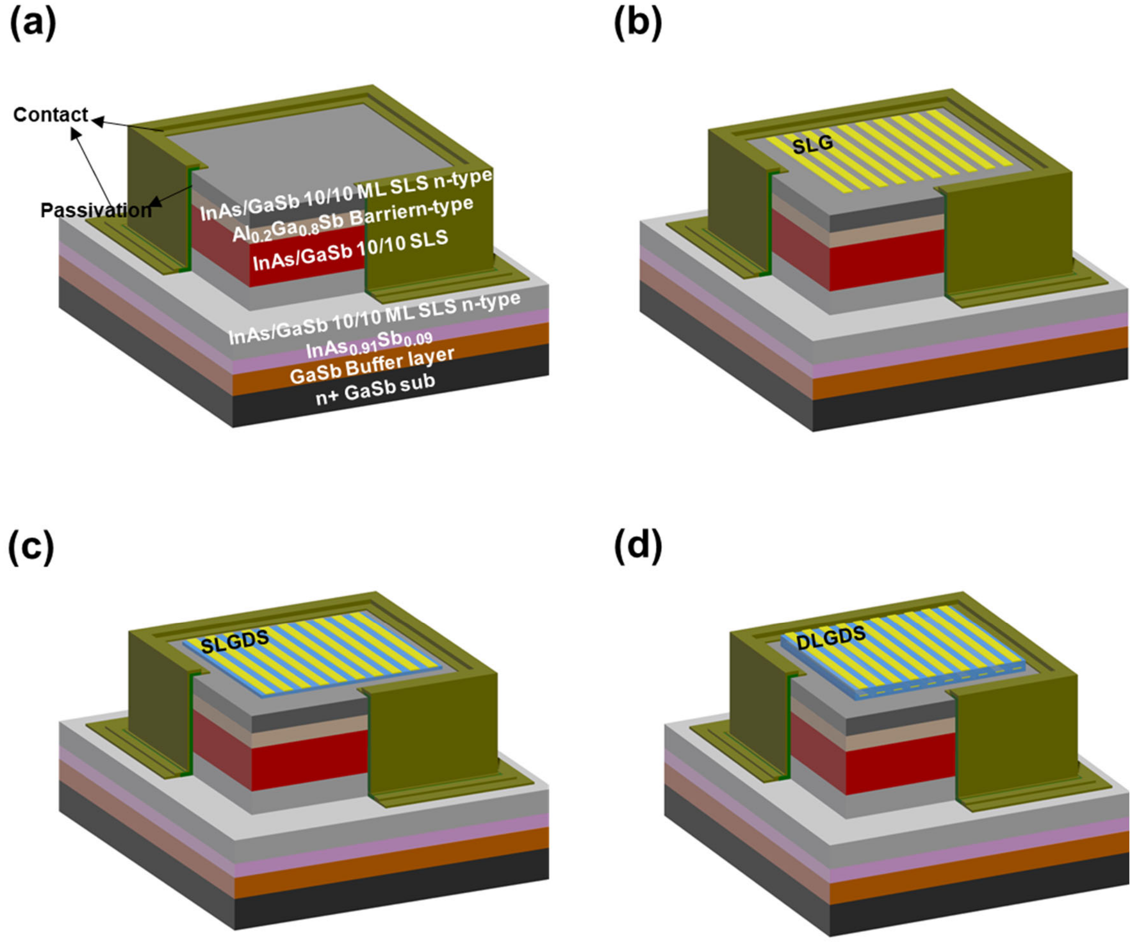

Supplementary figure S9 - Schematic illustration of (a) Bare device, (b) SLG device integration, (c) SLGDS device integration and (d)DLGDS device integration.

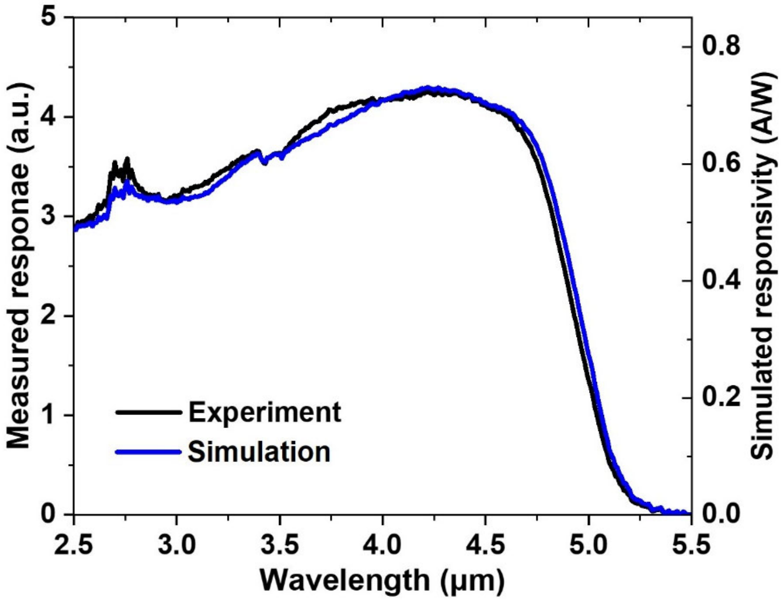

Supplementary figure S10. The simulated and measured spectral responsivity of T2SL bare device at 77K, bias voltage -1V.

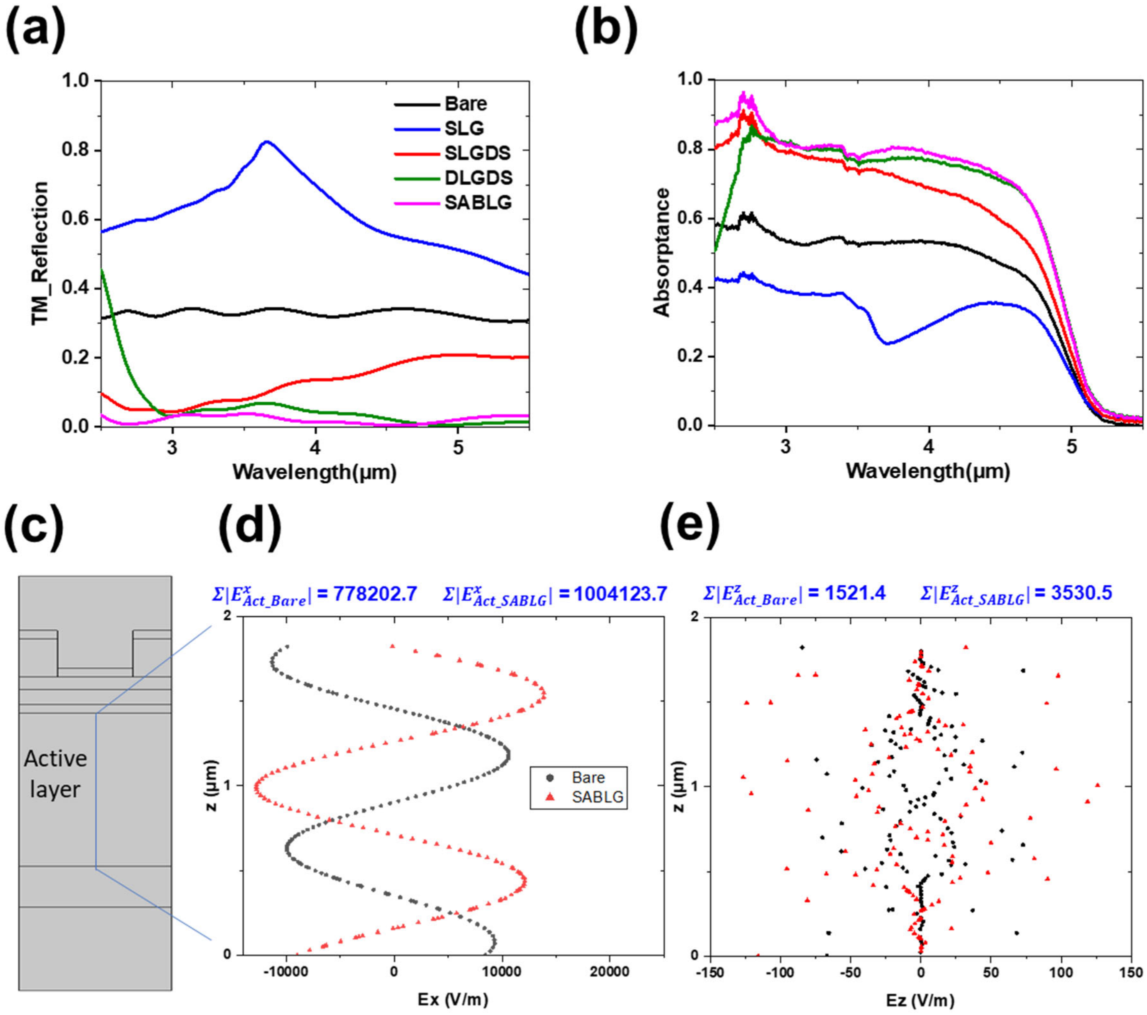

Supplementary figure S11. (a) TM reflection and (b) Absorption in the active layer for the bare T2SL and T2SL integrated with SLG, SLGDS, DLGDS, and SABLG. (c) Illustration of SABLG integrated T2SL device. (d) E_Act^x, (e) E_Act^z parallel to within an active layer at the center of the device (x=0) along z (0 μm ≤ z< 2 μm) at 4 μm wavelength.

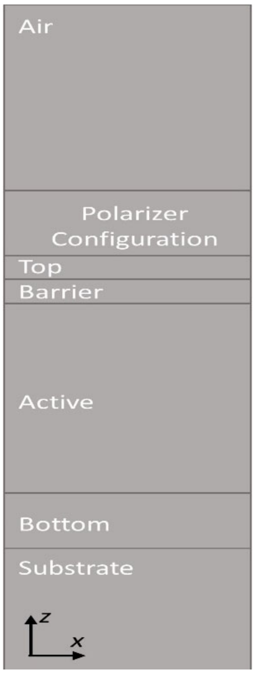

Supplementary figure S12. Schematic illustration of the simulation model of simplified T2SL device.
